# Focusing on the ethnobotanical uses of plants in Mersin and Adana provinces (Turkey)

**DOI:** 10.1186/1746-4269-1-6

**Published:** 2005-09-06

**Authors:** Ayse Everest, Ersin Ozturk

**Affiliations:** 1 Mersin University, Science &Art Faculty, Biology Department, Ciftlikkoy-Mersin, Turkey

**Keywords:** Ethno-botany, Medicinal plants, Mersin, Adana, TURKEY.

## Abstract

This paper presents the result of a study on the herbal drugs in the herbal markets in Mersin and Adana. The data were collected through direct interviews with herbalists and customers between 2002–2005 and the popular medicinal plants were investigated. A total of 107 species belonging to 56 families were investigated and the samples were listedwith their local and Latin names. The investigation includes cross-checking the disorders and their herbal cures and their recommended use stated by the local herbalists, by the parts used, and by the preparations. The cultivated species and their ethno botanical uses, are documented and extensive inventory is presented.

As a result, we observed that these plants are used especially for intestinal digestive disorders of the gastrointestinal tract, (21.68%), respiratory tract system disorders (10.43%), heart-blood circulatory system disorders (8.48%), urinary tract system disorders (7.70%), skin disorders (6.48%) and others.

## Background

In recent years, the increase in the residential and agricultural areas, and the decrease in medical plants have triggered the interest in ethno-botanical studies throughout the world [1-4]. The interest in herbal medicine in Turkey has progressed parallel to the increased interest in other developed countries. Recently, various studies have been conducted to prevent the folk medicine from disappearing [5-12].

For centuries, Turkish people have been using herbal medicine for the treatment of some daily diseases. The Taurus Mountains are one of the centers of the Mediterranean Region with a rich plant diversity. Accordingly, the traditional herbal medicines are important for the life of people. In this area, contagious diseases, cardiovascular disorders and cancer were investigated [13-18]. The world health report that provide us with the global rates and causes of mortality of cancer, contagious/parasitical diseases, circulatory system disorders, respiratory tract system and nerve disorders is presented in this study as a main source of analogy [19].

The aim of this, research is to focus on the kinds of medical diversity found in the herbal markets, on the frequency of usage of the plants, and thus, to show the different treatment types that are applied in the region.

The study is to be the first survey stating the herbal drugs in the herbal markets in the south of Turkey (Adana and Mersin provinces).

## Methods

Our chosen study area, The Taurus Mountains' hills are located in the south of Turkey with C4–5–6 grid squares. The population densities of the two central cities (Mersin and Adana) are 537, 842 and 807, 934 [20].

**Figure 1:**
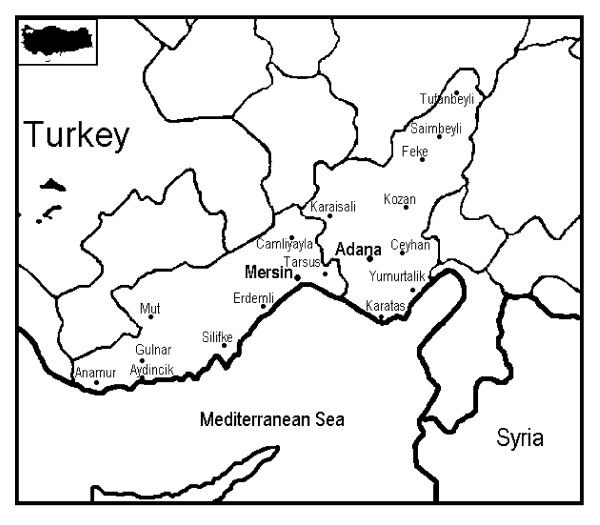
The Research Area.

There are about 70 herbal markets in the centers of Mersin and Adana. The plants presented in the herbal markets are collected from the villages in the Taurus Mountains (Figure 1). In the villages, dominant forest species are as follows: *Pinus brutia*, *P. nigra*, *Quercus coccifera*, *Q. cerris*, *Q. ithaburensis*, *Q. infectoria*, *Juniperus oxycedrus*, *J. excelsa*, *J. drupacea *and *Abies cilicica *[21,22]. The medical plants are harvested from places such as open areas, steppes, scrubs and roadsides. The plant materials are sold as dried bunches in open or pre-packed mixtures or as fresh preparations. The purchases depend on the request of the patient or on recommendation of the herbalists. Consumers generally boil these plants, make them into ointments or mix them with other plants depending on their intended use. The information about herbal medicine is gathered from at least two sources. The first source is the *oral folklore *that is passed on from one generation to the next and the second source is Ottoman, Arabic and Turkish *herbal books *which are sold in the bookstores.

During a two year-long survey, almost all herbal markets in the research area are investigated. The fresh plants and dried samples that are purchased from various herbal markets have been cross-examined with reference books [21-25]. The data were collected through direct interviews with herbalists and customers. Thirty herbalist and 10 customers were interviewed. The number of the recommended uses are 1732 in total. Among them 26 species are recommended by Mersin' s customers. The popular medicinal herbs used for treatments are shown to be monthly by low: 3 *, medium: 5 ** and high: 7 ***. A total 107 species (22 of them are cultivated) that belong to 56 families were investigated and the samples were listedby their local and Latin names by the treatment rates and by the medical health data given by WHO [26].

Some bio-reactive components are indicated by Evans [27] and Baytop [28].

Voucher specimens, in duplicates, are saved both in the Herbarium of Biology Department in Mersin and the Chemistry Department in Cukurova University.

## Results

While, *Origanum vulgare *L., *Micromeria myrtifolia *Boiss et. Hohen, *Teucrium polium *L., *Saturea hortensis *L., *Nepeta italica *L. and *Sideritis *spp. are used for making tea in the villages of the research area, nowadays the popular usage of tea plants such as *Rosa canina*, *Helichrysum stoechas*, *Myrtus communis*, *Sideritis congesta *and *Anthemis *spp. are used against obesity. The local people of Mersin ('yoruk') use *Helichyrsum stoechas *and *Hypericum perforatum *for stomachalgia, *Halimione portulacoides *for asthma, *Arum maculatum *for colitis, *Prunus avium, Myrtus communis *for obesity, *Mandragora officinarum, Ferula communis *for aphrodisiac, *Tussilago farfara *for cough, and *Capparis spinosa*, *Portulaca oleraceae*, *Crocus sativus*, *Juglans regia *for salad, pickle and jam.

According to the information obtained from some of the herbalists, the patients should start to accept the alternative therapies in health and medicine. Since diseases emerge due to the collapse of the immune system, the body should be cleaned up primarily. To achive this, it is suggested that a 3- month long therapy with *Urtica spp., Equisetum arvense *and *Achillea *spp. and a subsequent medical therapy would give good results.

## Discussion

A range from 7–33 species were from our list, have also been documented by several other researchers in a number of other countries [29-34]. In addition, about 30 plants in the list are declared in the synopsis of ESCOP and WHO Monographs on medicinal plants [35]. Except the diuretic plants, the uses of other plants are different in Turkey and Italy (such as; *Teucrium chamaedrys *for anti-malaria, *Laurus nobilis *for anti-stress, *Plantago lanceolata *for supporting and *Ocimum basilicum *for anti-headache in Italy [34]). Also, similar 23 species, 9 taxa, 41 taxa and 22 taxa in our list were reported by Turkish researchers [9,10,36,37].

From our list, taxa containing endemic species (*Thymus sipyleus *var.*sipyleus*, *T. cilicicus*, *Sideritis congesta *and *Liquidambar orientalis*)- are the only ones planted in the Tarsus by the East Mediterranean Forest Research Institute. These include Mediterranean elements (21.05 %), Euro-Sib. Elements (8.42 %), Ir.-Tur. Elements (5.26 %) and some widespread, widely cultivated plants (62.10 %) and several species like *Coriandrum sativum *and *Petroselinum crispum *which are unknown in origin according to Davis [21].

In Table 1 (see additional file 1) medicinal plants in the south of Turkey that belong to 56 families are listed below: Lamiaceae (13 sp.), Asteraceae (11 sp.) and Rosaceae and Apiaceae (8 sp.), Fabaceae and Brassicaceae (5 sp.), Poaceae and Urticaceae (3 sp.), Anacardiaceae, Iridaceae, Malvaceae, Moraceae, Polygonaceae, Solanaceae, Zygophyllaceae and Liliaceae (2 sp.). Other families are, Adianthaceae, Amaranthaceae, Araceae, Aspleniaceae and Berberidaceae (1 sp.), Boraginaceae, Buxaceae, Capparidaceae, Cupressaceae, Chenopodiaceae, Ericaceae, Equisetaceae, Fagaceae, Gentianaceae, Hamamelidaceae, Hypericaceae, Juglandaceae, Lauraceae, Linaceae, Loranthaceae and Lycopodiaceae (1 sp.), Myrtaceae (1 sp.), Onagraceae, Oleaceae, Orchidaceae, Papaveraceae, Pinaceae, Paeoniaceae, Plantaginaceae, Primulaceae, Plumbaginaceae, Portulacaceae, Rhamnaceae, Ranunculaceae, Resedaceae, Salicaceae and Tiliaceae and (1 sp.).

*Helichrysum stoechas *and *Anthemis *spp. (sigir papatyasi), *Reseda officinalis *and *Fumaria asephala *(sahdere), *Paliurus spina- christii *and *Lamium album *(ballibaba), *Hypnum cupressiforme *and *Lycopodium clavatum *(kurtpencesi), *Sideritis congesta *and *Salvia officinalis *(adacayi), *Cichorium inthybus *and *Taraxacum officinale *(hindiba), *Anthemis *spp. and *Matricaria chamomilla *(papatya), *Cupressus sempervirens *and *Quercus*, *Anagallis arvensis *and *Origanum majorona *(mercankosk) may be used for the same ailments with the same local names in some herbal markets of Adana. Locally, *Arbutus andrachne *is preferred instead of *Liquidamber orientalis *and some *Hypericum *spp. are preferred instead of *Centaurium erythrae *in Mersin. As various herbs can be advised to cure the same disease under one common name, to reach to the exact and right herbtype, and to prevent any misunderstanding or misusage of the herbal plants, herbalists and medical firms need to know the original Latin names of these herbs and ask accordinly before any purchase.

In the old herbal books *Gentiana lutea*, *Arum maculatum*, *Rumex acetosella*, *Nargissus *and *Opopanax *spp., were claimed to be abortive [38,39]. However, today, *Nasturtium officinale*, *Salvia officinalis*, *Nargissus*, *Cyclamen *spp, *Cinnamomum zeylanicum *(from India; tarcın) and *Cinchona officinalis *(from India; kına kına, kinin) are belived to be abortive and dangerous in herbal markets. In the research area these therapies were conducted either together with the medical therapy under physician control or in the cases that the medical therapy has failed.

In table 1, gunluk, dari, anason, ardic, papatya, sogan, meyan koku, mese, cemen, servi kozalagi, yabani roka, menengic, corek otu, defne, gul, semiz otu, kimyon, hindiba, gebere, keten, egrelti, sakiz, kisnis, mercankosk, nane, anason, ravent, sinirli ot, kekik, susen, sahdere, tere, yarpuz, safran was used in Central Asia according to Turk medicine books [40-45]. Today, these plants can be seen in Asian medicine treatments such as *Capparis spinosa*, *Apium graveolens*, *Equisetum arvense*, *Berberis vulgaris*, *Zea mays*, *Urtica dioica*, *Sambucus nigra*, *Capsella bursa-pastoris*, *Salix caprea *in Azerbaijan [46] and *Pteridium aquilinum*, *Myrtus communis*, *Plantago lanceolata*, *Portulaca oleraceae*, *Coriandrum sativum*, *Malva neglecta*, *Buxus sempervirens*, *Adianthum capillus-veneris*, *Mentha longifolia*, *Origanum vulgare*, *Nasturtium officinale *in Pakistan [47].

If we look at the XVI. Century medicinal plant list of the Ottaman Empire epoch, we can find the same list such as, adam otu, ada sogani, ahlat, adacayi, anason, andiz otu, ayva, bakla, baldıran, bogurtlen, biberiye, burcak, cakal erıgı, ceviz, corek otu, defne, dere otu, egrelti otu, feslegen, findik, funda, gul, guzel avrat otu, hardal, hindiba, incir, isirgan otu, karadut, keten, kekik, kedi otu, kıraz, kımyon, kuzukulagı, labada, lavanta, maydanoz, mercankosk, mersin, nane, zeytin, melisa, kantaron, kuskonmaz, misir puskulu, mese kabugu, ogul otu, pelin and safran, sakiz, sandal, sumak, susen [41,48-50]. Species that have been indicated above had been applied also in Central Asia and in the Ottoman-Turk Medical science. Even today they are still included on our list [44,45,51-53].

A great many of the vernacular names and common families and 15–24 species were shared in Anatolia and Central Asia [6,8,11,12,54]. For example, andiz/andiz for Inula sp., Quirkbog' um/kırkkilit otu for Equisetum sp., yarpuz/yarpuz for Mentha sp., Qoratut/dut for Morus sp., itburnu/kus burnu for Rosa sp., kılıchak/kılıc otu for Plantago sp., Asteraceae, Apiaceae, Lamiaceae and Rosaceae families (Table 1) [54].

In the light of these data, some plants that are presented in our list, can be said to be based on the Central Asia Turk medical science and also their prescriptions that had been used between the XIV-XVI Th. centuries.

It would not be inappropriate to say that, the similar climate and environmental conditions (especially in the regions of Mediterranean cultures) have been the cause of using the similar plants in that region [2,29,31,33,34], [54].

Table 2 shows clearly that in the south of Turkey (Mersin and Adana), the plants were used mainly for pathologies of the digestive, respiratory, heart-blood-liver, intestinal, urinary, skin system disorders, inflammatory and related ailments, nerve and related ailments and rheumatism, sprains and related ailments. These are followed by the others.

**Table 2:** The comparison with the other provinces and villages (Marmara region) in Turkey

**Pathologies**	**In Mersin, Adana (%) (Our study)**	**In Istanbul: Sile (%) [9]**	**In Balikesir: Gonen (%) [37]**	**In Sakarya (%) [36]**
Intestinal-digestive disorders	21.68	9.47	45.63	8.6
Respiratory system disorders	10.43	16.71	10.77	10.1
Heart-blood disorders	8.48	5.07	1.54	13
Urinary system disorders	7.70	7.99	9.23	9.4
Skin disorders	6.48	20.37	12.31	12.2
Antiinflammatory, antiseptic	6.20	3.63	4.61	-
Nerve disorders	5.73	-	-	13.7
Liver-spleen disorders	4.67	1.44	-	-
Gynecological disorders	4.42	0.72	-	2.9
Arthritis	3.16	7.26	7.69	1.4
Analgesic, anodyne, emollient	2.42	-	1.03	-
Sedative	1.90	-	1.53	2.9

## Conclusion

In conclusion, the comparison of the treatments between provinces and villages, shows us a decreased incidence of heart-blood, liver-spleen and gynecological disorders in the villages and increased incidence of the arthritis in the villages (Table 2) [9,36,37]. Contrary to the Mediterranean Region, there is an increased incidence of skin problems that are related to a humid climate and other different environmental conditions of the Marmara Region.

The percentage of skin disorders of Uzbekistan, Italy, Turkey and Greece implies that the increase may also be related to the technological developments, environmental pollution and humidity (Table 3).

**Table 3:** The comparison of the incidence of remedies between the other countries

**Pathologies**	**In Turkey (%) (Our study)**	**In Uzbekistan (%) [54]**	**In Italy (%) [33]**	**In Greece (%) [29]**
Intestinal-digestive disorders	21.68	31.20	10.60	16.93
Respiratory system disorders	10.43	13.30	13.00	3.06
Heart-blood disorders	8.48	5.10	1.35	7.93
Urinary system disorders	7.70	4.10	3.80	7.66
Skin disorders	6.48	16.60	11.50	4.32
Antiinflammatory, antiseptic	6.20	4.50	5.35	1.35
Nerve disorders	5.73	10.40	5.65	5.87
Liver-spleen disorders	4.67	8.10	2.56	5.22
Gynecological disorders	4.42	2.30	3.16	2.97
Arthritis	3.16	3.50	2.18	6.31
Analgesic, anodyne, emollient	2.42	-	1.27	-
Sedative	1.90	10.40	2.71	4.96

In Table 3, these results are interpreted with regional medical data; the first place in the list is taken by intestinal- digestive pathologies [13,14,17,55]. These results can be related to the fact that received immigration from the less developed cities and that they have rather poor hygienic conditions with regard to food and water [13,14]. However we have observed the same rates in Uzbekıstan [54], in Italy [32,33] and in Greece [29]. The World Health Report indicates that this problem appears in less developed countries of the world [19].

Nerve disorders are similarly seen in the Mediterranean countries as given in Table 3. Also, similar results are obtained -such as the percentages of rheumatism, digestive and respiratory system disorders- in Turkey and Uzbekistan.

In less developed countries, during childhood and adolescence, there is a factor of risk of contagious (infectious) diseases. The global mortality rate (over 52 million) depends on contagious and parasitic diseases (over 17 million), heart-blood system disorders (over 15 million), cancer (over 6 million) and chronic respiratory tract disorders (over 3 million) [19].

Following the listed results, we came up with the fact that the causes of mortality were mostly respiratory tract and circulatory system disorders [17,18], and that there is a low ratio of cancer (1.28 %) [16,55]. However, except cancer that is ranked as the third disease in the list of clinical world diseases, other rankings in our research findings seem identical/parallel with this list. This fact led us think that the herbal/cheap cures for cancer might have been deliberately exchanged with the chemical/expensive ones, or just carelessly overlooked.

In the end, the close analogy was discovered between the respiratory tract disorders, circulation system disorders, the intestinal-digestive diseases- which are related to the malnutrition, undeveloped hygiene habits, and the use of unnecessary antibiotics [56,57], and the medical health rates that are stated at the top three list of Turkey and the compared countries, drives our attention to the following herbs that can be used for all three health problems: *Origanum majorana*, *Equisetum arvense*, *Glycyrrhiza glabra*, *Matricaria chamomila*, *Nigella arvensis*, *Paliurus spina-christii*, *Armeniaca vulgaris*, *Linum catharticum*, *Orchis anatolica*, *Rosmarinus officinalis*, *Myrtus communis*, *Lavandula stoechas *and *Mentha pulegium*.

## Supplementary Material

Additional File 1 Table 1. The list of Medicinal plants of research area Click here for file
