# Supplementary material for: Focusing on the ethnobotanical uses of plants in Mersin and Adana provinces (Turkey)
Source: J Ethnobiol Ethnomed. 2005 Sep 6;1:6. doi: 10.1186/1746-4269-1-6 (PMC1277086; doi:10.1186/1746-4269-1-6)
Supplement: Additional File 1 — Table 1. The list of Medicinal plants of research area [file 1746-4269-1-6-S1.pdf]

TABLE 1. LIST OF THE MEDICINAL PLANTS RECORDED IN THE RESEARCH AREA

Botanical Families	Botanical name	Local name	Specimen no	Part(s) used	Constituents	Recommended uses by the herbalists/medicinal popular use: low (*), middle (**), high(***)	Preparations	Records
**Adianthaceae**	*Adiantum capillus-veneris* L.	Su sumbulu	E.1067/M	Lvs/M<br>Lvs/A	Tannin, musilaj	A,M:<br>Emmenagogue, gynecological disorders, diuretic (*)<br>A: Antitussive, expectorant, bronchial trouble (*)	Infusion<br>Vaporization	C1 (3)<br>C4 (5)<br>HDD (5)<br>T (5)<br>NLH (5)
**Amaranthaceae**	*Rumex crispus* L.	Labada<br>Kuzukulagi<br>Ravent	E.1390/M	Lvs/M<br>Lvs/A<br>Roots/A	Vitamin A, B, C, D, E, hydroxilic acids	A,M: Purgative, anti-inflammatory, antirheumatic, stomachic, diuretic, emmenagogue, antidiarrhoeal, febribuge, allergy, gingivitis, paludism(*)<br>A: Carminative, abortigenic, arteriosclerosis, (*)	Cataplasm<br>Decoction	T (3)<br>C4 (5)<br>NLH (7)<br>C2 (3)<br>KY (3)
**Anacardiaceae**	*Rhus coriaria* L.	Sumak	E.1376/M	Sds/M<br>Lvs/M<br>Sds/A	Tannin, sugar, flavone derivates, carotenoids	A,M: Antidiarrhoeal, hemostatic, antiseptic, digestive, orexigenic, antirheumatic, diaphoretic, chollagogue, antihemorrhoidal (***)<br>A: (***)	Direct application<br>Decoction	BC (5)
**Anacardiaceae**	*Pistacia terebinthus* L.	Sakizotu<br>Menengic	E.1392/M	Sds/M	Turpentine, dyeing and tanning materials, phenolic compounds, triterpenoid acids, alcohol	A,M: Diuretic, cardiotonic, asthma (*)	Decoction	BC (7)

**Apiaceae**	*Ammi visnaga* (L.) Lam.	Disotu Hiltan	E.1171/M<br><br><br>E1410/A	Sds/M<br><br><br>Frs/A	Visnagin, khellol glucoside, volatile oil	A,M: Spazmolytic, anthilitic, diuretic, antitussive, carminative, angiemphraxis, (**)<br>A: Kidneystones (*)	Direct application, Tooth-pick	C1 (3) T (7) NLH (7)
**Apiaceae**	**Apium graveolens* L.	Kereviz (Celery)	E.1373/M	Sds/M	Terpenes, anhydrine of sedadonic acid, phenols, coumarins, funarocoumarins, coumarin glycosides, lectin, apiin [1]	A,M: Anti-inflammatory, gynecological disorders (**)	Decoction Vaporizatio n	BC (1)
**Apiaceae**	**Anethum graveolens* L.	Dere otu	E.1356/M<br><br><br><br>E.1447/A	Sds/M Lvs/M<br><br><br>Flv/A Stem/ A	Carvone (55-70 %), limonene	A,M: Antiseptic, lactagogue, carminative, orexigenic, stimulant, eye inflammation, aphrodisiac (**)<br>A: Indigestion, goiter stress, bed-wetting (***)	Decoction	C1 (5) NLH (7) KY (5)
**Apiaceae**	*Coriandrum sativum* L.	Kisnis Zuhretaragi	E.1362/M<br><br><br>E.1431/A	Frs/M Lvs/M Sds/M SdsA	Linalool (65-80 % alcohols), terpenes	A,M: Orexigenic, stomachic, carminative, cardiotonic, liver disorders, blood stimulant, digestive hypertension (**)<br>A: Anodyne (**)	Direct application Pulverized/ M	C1 (5) T (5) KY (5)

**Apiaceae**	*Ferula communis* L.	Caksirotu<br>Cavsir otu	E.1363/M<br><br><br><br>E.1451/A	Sds/M<br>Roots/M<br>Flv/M<br>Stem/M<br>Sds/A<br>Flv/A<br>Lvs/A	Vitamins, carotens [3]	A,M: Antianemic, aphrodisiac (***)<br>A: Orexigenic, digestive, carminative, vermifuge (*)	Direct application	T (7)<br>NLH (7)<br>KY (7)
**Apiaceae**	*Foeniculum vulgare* Miller	Raziyane<br>Rezene<br>Mayana	E.1364/M<br><br><br><br><br><br>E.1414/A	Sds/M<br><br><br><br><br><br>Root/A<br>Lvs/A<br>Frs/A	Vol. oil, Anethole (60 %), fenchone, a ketone (20 %)	A,M: Stomachic, aromatic, carminative, lactagogue, diuretic, antirheumatic, antiinflammatory, antiseptic, urinary system disorders, antitussive, antilithic, adrenal agent, obesity, purgative (***)<br>A: Sedative, anodyne, sialogogue, heart stimulant, antidiarrhoeal (***)	Decoction	T (7)<br>NLH (7)<br>KY (3)<br>C1 (7)
**Apiaceae**	**Petroselinum crispum* (Miller) A. W. Hill.	Maydanoz	E.1406/M	Lvs/M<br>Stem/M	Coumarins, vol. oils, terpens<br>Petroselinum spp. : apiole (dimethoxysafrole)	A,M: Diuretic, expectorant, cancer, urinary system disorders (**).	Decoction<br>Infusion<br>Vaporization	BC (1)
**Apiaceae**	*Pimpinella anisum* L.	Anason	E.1373/M	Sds/M<br><br><br><br><br>Sds/A	Fixed oil, vol. oil	A,M: Orexigenic, carminative, lactagogue, anodyne, spazmolytic, digestive, diuretic, antiseptic, aromatic, tuberculosis, wounds, antitussive, aphonia, respiratory system disorders (**)<br>A: Emmenagogue (***)	Decoction<br>Paste	NLH (5)<br>KY (7)

**Araceae**	*Arum maculatum* L.	Agu,<br>Yilan yastigi<br>Yilana agu veren Mayasil otu<br>Esek kulagi<br>Buzagiotu	E.1359/M	Root/M<br>Lvs/M<br>Lvs/A<br>Root/A	Lectin, lipid, protein	A,M: Purgative, diaphoretic, antiinflammatory, stomachic, diuretic, bronchial trouble, antihemorrhoidal, psoriasis (*)<br>A: Orexigenic, digestive, lactogogue, carminative, sedative, pimples, cough, (**)	Cataplasm	C1 (3)<br>C4 (5)<br>NLH (5)<br>KY (3)
**Aspleniaceae**	*Asplenium ceterach* L.	Altınbasak	E.1454/A	Lvs/A	Volatile oil, tannin, musilage	A: Urinary system disorders (*)	Infusion	BC (1)
**Asteraceae**	*Achillea falcata* L.	Civan percemi<br>Civan percin<br>Kandil cicegi<br>Acelya	E.1354/M<br>E.1354/A	Flv/M	Alcohol, essential oils, salts, acids	A,M: Diaphoretic, antipyretic, diuretic, hypotensive, astringent, urinary system disorders, antiseptic, emmenagogue, gynecological disorders (***)	Infusion	C3 (5)<br>NLH (7)<br>KY (7)
**Asteraceae**	*Achillea nobilis L.*		E.1446/A	Flv/M<br>Lvs/A	Achillea spp. Essential oils, coumarins, sesquiterpenes	A,M: Allergy, antipruritic, psoriasis (***)<br>A: Carminative, digestive, cough, grippe (***)	Decoction	HDD (5)
**Asteraceae**	*Anthemis* spp.	Sigirpapatyasi<br>Sigir gozu<br>Okuz gozu	E.1357/M<br>E.1445/A	Flv/M	Anthemis nobilis: vol oil, n-butyl angelate, isoamly angelate, 3-phenylpropyl isobutyrate, tridecanal pentadecanal, terpenes	A,M: Diuretic, antirheumatic, ear disease, antineoplastic, antitussive, bronchial trouble, asthma (**)<br>A: Spazmolytic, carminative, antiinflammatory, ulcer, sedative, emmenagogue (***)	Decoction<br>Infusion	C1 (7)<br>C4 (5)<br>NLH (3)<br>KY (3)
**Asteraceae**	*Arctium tomentosum* Miller	Dulavrat otu<br>Bodurotu	E.1373/M<br>E.1423/A	Flv/M<br>Lvs/M<br>Root/M<br>Root/A	Vol. oil, tannin	A,M: Antiinflammatory, skin disorders, purgative, eczema, baldness (*)<br>A: Diaphoretic, expectorant, emmenagogue (***)	Decoction<br>Oil	C1 (7)<br>T (1)

**Asteraceae**	*Artemisia absinthium* L.	Acipelin	E.1358/M<br><br><br><br><br><br>E.1413/A	Flv/M<br>Lvs/M<br>Stem/M<br><br><br><br>Lys/A<br>Flv/A	Artemisia spp. santonin, volatile oil, crystalline lactone, artemisin	A,M: Orexigenic, digestive, diuretic, carminative, emmenagogue, anthelmentic, antidiarrhoeal, antianemic, antitussive, antiinflammatory, urinary- liver system disorders, diabetes (***)<br>A: Antiageing, febrifuge,(**)	Infusion<br>Decoction	T (7)<br>KY (3)<br>C1 (5)
**Asteraceae**	*Chrysanthemum coronarium* L.	Papatya (sarı papatya)	E.1461/A	Lvs/A<br>Root/A<br>Stem/A	Chrysanthemum spp. sesquitepene lactones, esters, polyphenols	A: Digestive, (**)	Decoction	BC (3)
**Asteraceae**	*Cichorium intybus* L.	Aci marul<br>Karahindiba<br>Hindiba	E.1375/M<br><br><br>E.1427/A	Flv/M<br>Lvs/M<br>Shoot/M<br>Lvs/A<br>Root/A	Inulin, sugars, coumarins, sesquiterpenes, lactones, flavone glycosides, vol. oil	A,M: Diuretic, stomachic, cardiotonic, liver disease, hepatoprotector, kidneystones (*)<br>A: Orexigenic, digestive, hematogenic, spleen, hearth ,liver disorders, rezolvent, pimples, antirheumatic (**)	Decoction	T (5)<br>C1 (5)
**Asteraceae**	*Helichrysum stoechas* (L.) Moench.	Sigirpapatyasi<br>Altinotu<br>Altinbasak<br>Olmezcicek	E.1376/M<br><br><br><br><br>E.1416/A	Flv/M<br>Whole/M<br>Plant/M<br><br>Flv/A<br>Lvs/A	Oil, tannin, resin, bitter, flavone derivates, cumarin	A,M: Antiinflammatory, antilithic, cough, bronchial trouble, asthma (***)<br>A: Diuretic, vulnerary, antihemorrhagic, antirheumatic, eczema (***)	Decoction	BC (5)

**Asteraceae**	*Inula helenium* L.	Andiz koku	E.1377/M		Helenin, alkaloid, inulenin, inulin, pigments, vol. oil [4]	A,M: Antitussive, asthma, expectorant, stomachic, diaphoretic, hepatitis, antiseptic, urinary system disorders, antipruritic, arthrosis (**)<br>A: Grippe, cough, respiratory system disorders (***)	Decoction	C3 (7)
**Asteraceae**	*Matricaria chamomilla* L.	Papatya	E.1388/M<br><br>E.1445/A	Flv/M<br><br>Flv/A	Sequiterpene, alcohols, ethers, oils, fatty acids, esters	A,M: Diuretic, purgative, diaphoretic, antilithic, aphrodisiac, cough, hair tonic (***)<br>A: Carminative, spazmolytic, antiinflammatory, emmenagogue,<br>obesity, baldness, digestive, heart, respiratory system disorders, stimulant (***)	Decoction	C1 (7)<br>T (7)<br>KY (7)
**Asteraceae**	*Tussilago farfara* L.	Farfara otu<br>Oksurukotu	E.1311/M	Lvs/M	Mucilage, bitter	A,M: Antitussive, respiratory system disorders (**)	Decoction	BC (3)
**Berberidaceae**	*Berberis crataegyna* D. C.	Kadin tuzlugu	E.888/M	Lvs/M	Tannin, organic acids, vitamin C	A,M: Stomachic (*)<br>A:Diabetes (**)	Decoction	C3 (7)
**Boraginaceae**	*Anchusa italica* Retz.	Sigir dili	E.1412/A	Stem/A	Essential oils	A: Cough, nervosity, tranquillizer, bronchial trouble, lactogogue, febrifuge (*)	Decoction	HDD (7)
**Brassicaceae**	*Alyssum saxatile* L.	Kuduz otu	E.1409/A	Root/A<br>Lvs/A	Essential oils, vit. A, C, B, D, K, phosphorus	A: Respiratory system disorders antiseptic, emetic, toothache (*)	Pulverized/A<br>Decoction	BC (1)

**Brassicaceae**	*Capsella bursa-pastoris* (L.) Medik.	Coban cantasi	E.1361/M	Frs/M<br>Flv/M<br><br>Flv/A<br>Lvs/A	Alcohols (methanol, ethanol, isopropanol), alpha-diazoester	A,M: Arthritis, antirheumatic, diaphoretic, hemostatic arteriosclerosis (**)<br>A: Diuretic ,antidiarrhoeal (***)	Decoction	C1 (7)<br>T (7)<br>NLH (7)<br>KY (3)
**Brassicaceae**	**Eruca sativa* Miller	Roka<br>Circir<br>Kekes	E.1391/M	Sds/M	Vitamins, minerals, proteins, fatty acids	A,M: Aphrodisiac, antirheumatic, hemostatic (*)<br>A: (***)	Direct application	C3 (7)<br>KY (3)
**Brassicaceae**	*Nasturtium officinale* R. Br.	Su teresi<br>Circir<br>Gerdeme	E.1433/A	Flv/A	Vitamin A,C, D, vol. oil, glykozides	A: Digestive, liver disorders (**)	Direct application	HDD (7)
**Brassicaceae**	*Sinapis arvensis* L.	Hardal<br>Yabani hardal	E.1278/M<br><br><br><br>E.1434/A	Flv/M<br>Frs/M<br>Sds/M<br><br>Sds/A<br>Lvs/A	Esters, alcohol, gelatin, acids, gum, starch, alginic acid and the like	A,M: Analgesic, adrenal agent, skin disorders, gingivitis, carminative, aphrodisiac, anthelmentic (**)<br>A: Purgative, urinary system disorders, liver disorders, antitussive, antirheumatic, sedative, bronchial trouble, toothache (**)	Decoction<br>Paste	T (5)<br>HDD (5)<br>NLH (5)<br>C1 (3)
**Buxaceae**	*Buxus sempervirens* L.	Simsir<br><br>Simsek agaci	E.1360/M<br><br>E.1448/A	Lvs/M<br>Flv/M<br>Lvs/A<br>Root/A<br>Bark/A	Resin, vol. oil, gum, akaloids	A,M: Headache, liver disease (*)<br>A: Diaphoretic, febrifuge, diuretic, vermifuge, hair tonic (*)	Decoction	T (3)<br>NLH (3)<br>KY (3)<br>C1 (3)

**Capparidaceae**	*Capparis spinosa* L.	Teber<br>Kebere	E.1277/M	Frs/M<br><br>Frs/A<br>Flv/A	Alks-stachydrine, glucocapparin flavonoids, sterols, terpenes	A,M: Stomatitis, spleen, heart, liver disease, baldness, digestive, aphrodisiac (***)<br>A: Diuretic, antidiarrhoeal (*)	Pickle	C1 (1)<br>HDD (3)
**Chenopodiaceae**	*Halimione portulacoides* (L.) Aellen	Kaya korugu<br>Aci dam korugu	E.1393/M	Lvs/M	[5]	A,M: Diuretic, antidiarrhoeal, intestinal disorders, goiter (**)<br>A: Headache (*)	Decoction, Cataplasm<br>Pickled fresh materials	BC (7)
**Cupressaceae**	*Juniperus drupacea* Lab.	Andiz	E.1054/M<br><br>E.1417/A	Frs/M<br>Sds/M<br><br>Frs/A	Juniperus spp.: oil, sesquiterpenes, phenolic compounds	A,M: Bed-wetting, arthrosis, tuberculosis, hearth stimulant, emmenagogue, asthma, antirheumatic, aphrodisiac, adrenal agent (***)<br>A: Decrease effect of cigarets (***)	Jam	C4 (5)
**Equisetaceae**	*Equisetum arvense* L.	Kirkkilit otu<br>Atkuyrugu<br>Zemberekotu	E.1047/M	Lvs/M<br>Stem/M<br>Lvs/A	Saponins, silisilik acid, tannin, alkaloids	A,M: Diuretic, anthilitic, hemostatic, antiinflammatory, collagogue, diaphoretic, cancer, emollient, expectorant, stomachic, urinary system disorders, disinfectant, antirheumatic, hepatoprotector, antianemic, allergy, asthma (***)<br>A: (***)	Infusion	C1 (7)
**Ericaceae**	*Erica manipuliflora* Salisb.	Funda<br>Supurge otu	E.1147/M<br><br>E.1444/A	Lvs/M<br>Flv/M<br><br>Lvs/A	Vol. oil, phenolic acids	A,M: Diuretic, obesity, lactagogue, antidiarrhoeal, depurative, hypercholesterolemia (***)<br>A: (***)	Infusion	T (7)<br>C1 (7)<br>NLH (7)<br>KY (7)<br>C1 (7)
**Fabaceae**	**Cicer arietinum* L.	Nohut	E.1401/M	Sds/M	Lectin.	A,M: Aphrodisiac, diaphoretic, antirheumatic, orexigenic (**)<br>A: Anodyne (*)	Cataplasm, Direct application	

**Fabaceae**	*Glycyrrhiza glabra* L.	Meyan koku	E.1366/M<br>E.1452/A	Root/M<br>Root/A	Glycrrhizin, glycrrhizinic acid, flavonoids, liquiritin, isoliquertin, and other compounds, volatile compounds, stach, protein	A,M: Expectorant, antitussive, asthma, aphonia, anacatharsis, antiinflammatory, antidepressant, purgative, stethalgia, antiseptic, urinary system disorders, constipation, antianemic, kidneystones (***)<br>A: Ulcer, febrifuge, hypotension (***)	Decoction	C1 (7)<br>T (7)<br>NLH (7)<br>KY (7)
**Fabaceae**	**Lathyrus sativus* L.	Burcak	E.1367/M	Sds/M<br>Sds/A	Stearic acid, thiamine, niacin, vit. B 12, E	A,M: Diuretic, digestive, gingivitis, arthrosis, eczema, cancer, stomachic, diabetes (*)<br>A: Aphrodisiac, ulcer (*)	Decoction<br>Pulverized/M	C1 (3)<br>NLH (7)<br>KY (3)
**Fabaceae**	*Trigonella coerulescens* (Bieb.) Hal.	Cemen<br>Boyotu<br>Cimen	E.1394/M<br>E.1436/A	Frs/M<br>Sds/M	Resin, vitamins, vol. oil, proteins, saponin	A,M: Aphrodisiac, diuretic, expectorant, asthma, antihemorrhoidal, antilithic, aromatic, arthritis (**)<br>A: (**)	Decoction<br>Pulverized/A	HDD (7)
**Fabaceae**	**Vicia faba* L.	Aci bakla<br>Bakla	E.1385/M	Frs/M	Resin, tannin, citric acid, vitamin E, tannic acid	A,M: Psilotic, diuretic, diabetes (**)<br>A: Depurative, sedative (***)	Cataplasm	HDD (7)<br>KY (5)
**Fagaceae**	*Quercus* spp.<br>*Q. coccifera* L.<br>*Q. infectoria* Olivier	Palamut<br>Pelit<br>Pirnal	E.1199/M	Bark/M<br>Frs/M<br>Frs/A	Tannin, gallic acid, egallic acid, sitosterol, methyl betulate, methyl olearolate, starch, calcium oxalate, nyctanthic roburic and syringic acids	A,M: Bullation, algia, antineoplastic, antilithic, cancer, stomachic (*)<br>A: Gonorrheal, antihemorrhoidal, purgative, antidiarrhoeal (*)	Decoction	C3 (3)<br>NLH (3)<br>KY (3)

**Gentianaceae**	*Centaurium eryhtrae* Rafn.	Kirmizi kantaron	E.1135/M<br>E.1029/M	Flv/M<br><br>Flv/A<br>Lvs/A<br>Frs/A	Vol. oil, resin, bitter	A,M: Orexigenic (**)<br><br>A: Indigestion, wounds, spazmolitic, ulcer, sedative, antiseptic (***)	Decoction	T (7)<br>C1 (3)
**Hamamelidaceae**	*Liquidambar oriantalis* Miller	Gunluk	E.1369/M<br><br><br>E.1432/A	Br/M<br><br><br>Root/A	Cinnamic acid (balsamic acids), phenylethylene (styrene), cinnamic esyers, vanillin free cinnamic acid, monoterpenes, phenylpropanes, aliphatic acids	A,M: Antiseptic, fumigant, psychotonic, mnemasthenia, stammer (**)<br>A: Disinfectant, vulnerary, expectorant, nervosity, emollient (***)	Vaporization	T (7)<br>NLH (5)<br>KY (5)<br>C1 (7)
**Hypericaceae**	*Hypericum perforatum* L.	Kantaron<br>Binbirdelikotu	E.1293/M<br><br><br><br><br>E.1442/A	Flv/M<br><br><br><br><br>Flv/A	Anthraquinones; hypericin, pseudohypericin, prenylated phloroglucinol phloroglucinol derivates; hyperforin, adhyperforin, furohyperforin, flavonoids, volatile oil, vitamin C, tannins, carotenoids, aminoacids	A,M: Spazmolytic, antidiarrhoeal, sedative, antiseptic, stomachic hepatitis, hemostatic, vulnerary, chollagogue, hepatoprotector, antiinflammatory (***)<br>A: Orexigenic, digestive (***)	Decoction<br>Infusion	C1 (7)<br>T (7)<br>C3 (5)
**Iridaceae**	**Crocus sativus* L.	Safran<br>Aspir	E.1400/M	Flv/M<br>Root/M<br><br><br><br>Flv/A	Vol. oil, caretinoid pigments, crocin, terpene, esters	A,M: Cardiotonic, stomachic, tranquillizer, mouth inflammations, antitussive, aphrodisiac, orexigenic, emmenagogue (**)<br>A: Ophthalmitis, toothache (***)	Direct application<br>Pulverized/M	T (3)<br>NLH (5)<br>KY (7)<br>C1 (7)
**Iridaceae**	*Iris pseudacorus* L.	Guz cigdemi<br>Sari susen	E.1399/M	Root/M	Alcohol, oil, metyl esters of fatty acids	A,M: Hemostatic, antiia, ambustion, fire burn, psilotic	Direct application	HDD (5)

						(**)<br>A: Gynecological disorders (*)	Pulverized/<br>M	
**Juglandaceae**	**Juglans regia* L.	Ceviz	E.1150/M	Frs/M<br>Pods/<br>M	Lectin, oil, mineral, aminoacids, proteins, vitamins	A,M: Orexigenic, stomachic, carminative, antiseptic, antirachitic, antipoison, antipruritic, antiinflammatory, antihemorrhoidal, vermifuge, asthma (***)<br>A: Hypercholesterolemia, antidiarrhoeal (***)	Direct applications	C3 (7)<br>HDD (7)
**Lamiaceae**	*Ajuga chamaepitys* (L.) Sch.	Teyrek<br>Mayasil otu<br>Hamam otu<br>Yer selvisi<br>Yer cami	E.1095/M	Lvs/M	Volatile oil, resin, bitter	A,M: Psilotic, ointment, exanthema, eczema (**)<br>A: (***)	Cataplasm<br><br>Pulverized/A	T (5)<br>HDD (3)
**Lamiaceae**	*Lavandula stoechas* L.	Karabas otu<br>Lavanta	E.1368/M<br><br>E.1419/A	Flv/M<br>Lvs/M<br><br>Lvs/A	L. officinalis : linalool, linalyl acetate, ethyl-perityl ketone	A,M: Nervosity, stethalgia, epilepsy, headache, anodyne, heart, blood stimulant, nervosity, hypertension, throat disorders (***)<br>A: Digestive, obesity (***)	Decoction	NLH (7)<br>KY (5)
**Lamiaceae**	*Melissa officinalis* L.	Ogulotu<br>Limonotu<br>Turuncubile	E.1108/M<br><br>E.1422/A	Lvs/M<br><br>Lvs/A	Glycosides of luteolin, quercetin, apigenin, kaempferol, hydroxycinnamic acids, caffeic, chlorogenic, rosmarinic acid	A,M: Tranquillizer, antidepressant, anhypnia, gastrit, mnemasthenia, angiemphraxis, epilepsy, fainting, allergy, digestive, cardiotonic (***)<br>A: Hearth stimulant,carminative, sedative, spazmolytic, diaphoretic, disinfectant (***)	Decoction<br>Infusion	T (7)<br>HDD (7)

**Lamiaceae**	**Mentha longifolia* (L.) Hudson	Nane	E.1119/M	Lvs/M	Essential oils, terpenoids	A,M: Stomachic, carminative, antineoplastic, aphrodisiac, vermifuge (***)<br>A: Diabetes, grippe (***)	Direct application<br>Decoction<br>Direct application	T (3)<br>C3 (5)<br>NLH (5)<br>C2 (3)<br>KY (5)<br>C1 (7)
**Lamiaceae**	*Mentha pulegium* L.	Yarpuz	E.1370/M	Lvs/M<br>Flv/A<br>Lvs/A	Essential oils, esters, stearic acid, alcohol, vol. oil	A,M: Hearth stimulant, gingivitis, antihemorrhagic, cardiotonic, carminative, respiratory system disorders, diabetes, emmanagogue (**)<br>A: Orexigenic, digestive, expectorant(**)	Decoction<br>Cataplasm	T (3)<br>C3 (5)<br>NLH (5)<br>C2 (3)<br>KY (5)
**Lamiaceae**	*Micromeria myrtifolia* Boiss&Hohen	Dagcayi	E.1114/M	Stem/M	Vol. oil	A,M: Stomachic, throat disorders, bullation (*)<br>A: Grippe, stomatic (***)	Decoction	C3 (7)
**Lamiaceae**	**Ocimum basilicum* L.	Reyhan<br>Feslegen	E.1371/M<br>E.1439/A	Lvs/M<br>Sds/M<br>Lvs/A	Vol. oil, terpenoids, saponins, alkaloids	A,M: Purgative, antidepressant, vulnerary, antirheumatic, antidiarrhoeal (***)<br>A: Orexigenic, digestive, carminative, antiinflammatory (***)	Infusion<br>Direct application	T (7)<br>NLH (7)
**Lamiaceae**	*Origanum majorana* L.	Feslegen<br>Balikotu<br>Mercankosk<br>Merzacus<br>Merdegus<br>Gelinguvey cicegi<br>Bilyeli kekik	E.1387/M	Lvs/M<br>Lvs/A<br>Stem/A	Vol. oil, terpenoids, tannins, alkaloids	A,M: Bed-wetting, sedative, disinfectant, carminative, sudoral, antidiarrhoeal, urinary system disorders, abepithymia, cirrhosis, grippe, bronchial trouble, diuretic, digestive, analgesic (***)<br>A: Orexigenic, spazmolytic, antitussive, gingivitis, indigestion, emmenagogue, asthma, obesity antirheumatic,	Infusion<br>Decoction<br>Direct application<br>Oil/M	KY (5)<br>C2 (3)<br>C4 (3)

						antiinflammatory, (***)		
**Lamiaceae**	*Rosmarinus officinalis* L.	Biberiye<br>Hamisebahar<br>Kusdiliotu	E.1377/M<br><br><br><br><br>E.1453/A	Lvs/M<br><br><br><br><br>Lvs/A<br>Flv/A	Borneol, linalool (10-18 %), bornyl acetate, terpenes, cineole	A,M: Asthma, epilepsy, bullation, anhypnia, hepatitis, spleen disorders, cephalalgia, bronchial trouble, digestive, hypercholesterolemia, antidiarhoeal, respiratory system disorders (***)<br>A: Stimulant, psychotonic, antiageing, orexigenic, febrifuge, diuretic, carminative, vulnerary, antirheumatic, hair tonic, hypertension (***)	Infusion<br>Decoction	C1 (9)<br>T (7)<br>KY (7)
**Lamiaceae**	*Salvia fruticosa* Miller	Adacayi	E.1456/A	Lvs/M<br>Lvs/A	Salvia spp. volatile oil, thujone, cineole, borneol	A,M: Prostate, liver disorders, ulcer, digestive, orexigenic(**)<br>A: Sedative, antiseptic, carminative, spicery, antiageing (**)	Decoction	T (7)<br>KY (7)<br>C1 (9)

**Lamiaceae**	*Sideritis congesta* P.H. Davis & Hub.-Mor.	Adacayi<br>Dagcayi<br><br>Ballibaba	E.1379/M<br><br><br>E.1418/A	Flv/M<br>Frs/M<br><br>Lvs/A<br>Flv/A	Vol. oil, tannin, bitter	A,M: Disinfectant, carminative, stimulant, regulatory hormone, antitussive, orexigenic, conservative, cough (**)<br>A: Digestive, diuretic, emmenagogue, abortigenic, sedative, hypoglicemian, antiageing (***)	Infusion<br>Decoction	BC (7)
**Lamiaceae**	*Teucrium polium* L.	Yavsan	E.1428/A	Lvs/A<br>Stem/A	Vol. oil	A: Urinary system disorders, diabetes, eczema (**)	Decoction	BC (7)
**Lamiaceae**	*Thymus* spp.<br>*T. sipyleus* Boiss,<br>*T. cilicicus* Boiss & Bal.	Kekik	E.1024/M<br>E.117/M<br>E.1435/A	Flv/M<br>Lvs/M<br>Stem/M	Thymus spp. thymol<br>Terpenoids, saponins, alkaloids	A,M: Hypotension, remedy alcohol poisoning (***)<br>A: Emollient, toothache (***)	Decoction<br>Direct application	T (7)<br>HDD (7)<br>NLH (7)<br>C1 (7)
**Lauraceae**	*Laurus nobilis* L.	Defne	E.1378/M<br><br><br>E.1443/A	Lvs/M<br><br><br>Lvs/A<br>Frs/A<br>Root/A	Essential oils	A,M: Diuretic, antirheumatic, orexigenic, digestive, carminative, stomachic, asthenic, artrosis, diaphoretic, antilihic, antidiarrhoeal, anacatharsis, baldness, emollient, tranquillizer (**)<br>A: Disinfectant, bronchial trouble, anodyne , hair tonic (***)	Decoction<br>Direct application	C1 (7)
**Liliaceae**	**Allium cepa* L.	Sogan	E.1402/M	Lvs/M<br>Bulb/M<br>Pods/M	Fructan diosynthetic enzymes, beta-amino-alpha-cyanoacrylates, lectin, carboxylamides, oil	A,M: Antiinflammatory, carminative, purgative, gynecological disorders, emmenagogue, obesity(**)<br>A: Orexigenic, indigestion, disinfectant, hypoglycemian,	Cataplasm<br>Infusion	C1 (7)

				Lvs/A<br>Stem/<br>A		diuretic, wounds (***)		
**Liliaceae**	*Urginea maritima* (L.) Baker	Ada sogani	E.1383/M<br>E.1458/A	Lvs/M	Glucoscillaren A, glycosides, flavonoids, sinistrin, inulin, mucilage, antocyanins	A,M: Vulnerary, ophidism, antirheumatics (**)<br>A: (*)	Cataplasm	KY (7)
**Linaceae**	**Linum catharticum* L	Keten<br>Zeyrek<br>Cayirkeneviri	E.1264/M<br>E.1420/A	Sds/M<br>Sds/A	Fatty acids, esters, potassium	A,M: Tranquillizer, demulcent, purgative, antitussive, emollient, anodyne, resolvent, allergy, antirheumatic, cardiotonic, urinary system disorders, digestive, antiinflammatory, antiageing, heart- brain hemorrhage (***)<br>A: Stomachic (***)	Oil	C1 (7)
**Loranthaceae**	*Viscum album* L.	Okseotu<br>Gokceotu	E.1153/M<br>E.1429/A	Lvs/M<br>Stem/<br>M<br>Sds/M<br>Lvs/A	Resin, saponins, acids, alkaloids, cyclitols, glycoproteins, polypeptides, lignans	A,M: Hypotensive, heart stimulant, sedative, antitussive, antineoplastic, remedy pruritus (***)<br>A: Diuretic,hypertension, spazmolytic, emetic ***	Cataplasm	T (7)<br>NLH (7)<br>C1(7)
**Lycopodiaceae**	*Lycopodium clavatum* L.	Aslan pencesi	E.1355/M<br>E.1408/A	Lvs/M	Fixed oil, glycerides of lycopodium oleic acid, sugars, phytostein, alkaloids	A,M: Lactagogue, vulnerary, febrifuge, headache (**)<br>A: Stomatitis(*)	Infusion<br>decoction	T (5)<br>KY (5)
**Malvaceae**	*Althaea officinalis*	Hatmi<br>Murver<br>Melekotu<br>Ebegumeci	E.1411/A		Mucilage; glucans, arabinan, polysaccharide, galactose, rhamnose,	A,M: Digestive, expectorant, ulcer (**)<br>A: Diuretic, antilithic, emollient, demulcent (**)	Decoction	BC (7)

					galacturonic acid, glucuronic acid, aspartic acid			
**Malvaceae**	*Malva sylvestris* L.	Ebegumeci	E.1271/M<br><br><br>E.1421/A	Lvs/M<br>Flv/M<br><br>Lvs/A<br>Flv/A	Aminoacids, alcohols, liquid fats, oils, waxes, esters, sugars	A,M: Anodyne, cancer, antineoplastic, antitussive, stomachic, algia, anacatharsis, throat disorders (*)<br>A: Aphonia, bronchial trouble, antiinflammatory, abscess (**)	Decoction	T (3)<br>C1 (5)
**Moraceae**	**Ficus carica* L.	Incir	E.1403/M	Frs/M<br><br>Lvs/A<br>Frs/A	Latex, vitamin A, B, C, D, E enzymes, essential fatty acids, minerals	A,M: Antitussive, gingivitis, hepatoprotector (*)<br>A: Sedative, expectorant (***)	Direct application<br>Juice/A	C1 (7)
**Moraceae**	**Morus nigra* L.	Dut	E.1404/M	Frs/M<br>Lvs/M	Flavanoides, caretinoids, vit. B, C, K1, triterpenes, sterols, salt, acids, sugar	A,M: Hypoglycemian, tranquillizer, cardiotonic, exanthema, blood stimulant, orexigenic (***)<br>A: (***)	Decoction<br>Jam	T (7)<br>C3 (7)<br>HDD (7)
**Myrtaceae**	*Myrtus communis* L.	Mersin<br>Murt	E.1178/M<br><br><br><br><br><br>E.1441/A	Lvs/M<br><br><br><br><br><br>Lvs/A<br>Frs/A	Tannin, vol. oil, bitters	A,M: Antidiarrhoeal, antiseptic, orexigenic, stomachic, diuretic, cardiotonic, vulnerary, antilithic, baldness, disinfectant, antihermorrhagic, urinary system disorders, respiratory system disorders, obesity (***)<br>A: Purgative, disinfectant, antiinflammatory, digestive (***)	Decoction<br>Infusion	T (7)

**Oleaceae**	* *Olea europaea* L.	Zeytin	E.1405/M	Sds/M Lvs/M<br><br>Lvs/A	Olive oils, olein, linolein, volatile C6 alcohols, C6 aldehydes and acetylated esters	A,M: Cancer, mnemasthenia, vulnerary, phymatosis, ambustion, pimples (***)<br>A: Diabetes (**)	Direct application Oil/M	BC (7)
**Onagraceae**	*Epilebium angustifolium* L.	Yaki otu	E.1144/M<br><br>E.1455/A	Lvs/M<br><br>Lvs/A	Hormones, vitamin C, E, K, esters, acid, gum arabic	A,M: Epilepsy, antipruritic (*)<br>A: Prostate (**)	Cataplasm	BC (3)
**Orchidaceae**	*Orchis anatolica* Boiss.	Salep	E.1366/M E.900/M	Root/M<br><br>Root/A	Sugar, musilage	A,M: Expectorant, emollient, cardiotonic, aphrodisiac, emmenagogue, palsy, bronchial trouble, vermifuge (***)<br>A: Nutrient, stimulant, antidiarrhoeal (***)	Direct application Pulverized/M	BC (7)
**Paeoniaceae**	*Paeonia mascula* Miller	Sakayik Ayigulu Dedegulu	E.1457/A	Flv/A Root/A	Tannin, vol. oil	A: Purgative, stomachic, chollagogue, hepatoprotector, urinary system disorders, treat joandice, hemagogue (*)	Direct application Decoction	BC (3)
**Pinaceae**	**Pinus pinea* L.	Fistikcami	E.1051/M	Shoots/M Bark/M Exudates/M Sds/M	Tanning materials Pinus spp: Bornyl acetate, terpenes, sesquiterpenes	A,M: Lactogogue, tuberculosis, mnemasthenia, apocamnosis, anodyne, expectorant, antihemorrhoidal, vulnerary (**)	Direct application Paste	NLH (7)
**Plantaginaceae**	*Plantago major* L.	Sinirli ot<br><br>Baldirikara	E.1068/M<br><br>E.1426/A	Lvs/M Stem/M<br><br>Lvs/A	Mucilage, tannin	A,M: Gingivitis, hepatitis, vulnerary, asthma, callus, analgesic, urinary system disorders, antibiotic, sedative (***)<br>A: Nervosity, grippe, cough (***)	Decoction	NLH (7) KY (5)
**Poaceae**	*Cynodon dactylon* (L.) Pers.	Ayrik otu	E.1407/A	Frs/A Flv/A	sodium carboxymethyl cellulose, ethyl cellulose and cellulose acetate;	A: Diuretic, blood stimulant, emollient, prostate (***)	Decoction	BC (3)

					powdered tragacanth; malt; gelatin; talc; excipients such as cocoa butter and suppository waxes; oils such as peanut oil, cottonseed oil; safflower oil; sesame oil; olive oil; corn oil and soybean oil; glycols; such a propylene glycol; esters			
**Poaceae**	**Triticum vulgare* L.	Bugday	E.1396/M	Sds/M	Lectin, fatty acids, lecithins, gummi arabicum, gelatine	A,M: Nutrient, brain and eyes disorders, hematogenic, tranquillizer, stomachic (**)	Cataplasm Mask	KY (5)
**Poaceae**	*Zea mays L.	Misir puskulu	E.1386/M<br>E.1440/A	Frs/M	Oleic, linoleic acids, triglycerides of the palmitic, stearic acids	A,M: Diuretic, antilithic, antiinflammatory, gout, antirheumatic, hepatoprotector, obesity (***)<br>A: Kidney stones (**)	Decoction	T (7)<br>C3 (5)<br>NLH (7)<br>KY (5)
**Polygonaceae**	*Polygonum aviculare* L.<br>*P. bistorta* L.	Ciyancik<br>Kurtpencesi<br>Encubar<br>Yilankoku<br>Kusekmegi<br>Madimak<br>Potuk<br>Yesil subiberi	E.1374/M	Lvs/M<br>Flv/M<br>Lvs/A	Tannin, flavone	A,M: Antihemorrhoidal, anacatharsis, obesity, antirheumatic, hemostatic, diaphoretic, hypotension, stomachic,<br>kidney stones, liver disease (*)<br>A: Baldness (**)	Infusion	KY (5)
**Portulacaceae**	*Portulaca oleracea* L.	Semizotu<br>Pirpirim<br>Tokmakan<br>Sogukluk	E.1236/M	Lvs/M	Fixed oil containing b-sitosterol,<br>(-)--noradrenaline, antioxidants	A,M: Sphagitis, bronchial trouble (*)<br>A: Stimulant (**)	Direct application	NLH (7)
**Primulaceae**	*Anagallis arvensis* L.	Suluk otu	E.1395/A	Lvs/A	Vol. oils	A: Urinary system disorders (*)	Decoction	T (3)<br>C4 (3)<br>NLH (3)

								KY (3)
**Ranunculaceae**	*Nigella arvensis* L.	Corekotu<br>Siyahsusam	E.1296/M	Sds/M<br>Sds/A	Fixed oil, alkaloids	A,M: Diuretic, lactogogue, orexigenic, emmenagogue, cardiotonic, headache, epilepsy, grippe, digestive, baldness, carminative, stomachic (**)<br>A: (***)	Oil	T (7)<br>HDD (7)
**Resedaceae**	*Reseda lutea* L. or *Fumaria asephala* Boiss. (Papaveraceae)	Sahdere	E.1365/M<br>E.1415/A	Lvs/M<br>Flv/M<br>Stem/M	Vitamin A, resin, OMEGA<br>Essential oil	A,M: Diuretic, depurative, angiemphraxis, tranquillizer, cholecystic, anacatharsis, eczema, hepatoprotector, antipruritic, allergy (**)	Infusion<br>Cataplasm	BC (5)
**Rhamnaceae**	*Paliurus spina-christii* L.	Ballibaba<br>Kusekmegi	E.1389/M	Frs/M<br>Lvs/M<br>Root/A<br>Lvs/A<br>Flv/A	Tannin	A,M: Prostate, antiinflammatory, urinary system disorders, ia, mucosa in throat disorders, antihemorrhoidal, diaphoretic, hematogenic (**)<br>A: Diuretic, hemostatic, gynecological disorders, antianemic, liver disorders, asthma, bronchial trouble (***)	Infusion<br>Cataplasm	BC (5)
**Rosaceae**	**Armeniaca vulgaris* Lam.	Kayisi	E.1374/M	Frs/M<br>Lvs/A<br>Frs/A<br>Sds/A	Oil acids, gelatine, sitosterol, lecithin, chitosans, fatty acids	A,M: Orexigenic, digestive, diuretic, carminative, emmenagogue, anthelmentic, antidiarrhoeal, antianemic, antitussive, antiinflammatory, urinary system desorders, constipation, hematogenic(***)	Direct application, Jam	C1 (7)<br>HDD (7)

						A: Purgative, (***)		
**Rosaceae**	*Agrimonia eupatoria* L.	Koyun otut	E.1371/M	Lvs/M<br>Flv/M<br>Flv/A	Fatty acids, essential oils, esters, salts	A,M: Antiinflammatory, hemagogue, emmenagogue, antilithic (*)<br>A: Indigestion, antidiarrhoeal, vulnerary, antirheumatic,aphonia, throat disorders (***)	Infusion<br>Decoction	T (3)<br>C4 (5)<br>C1 (5)<br>HDD (5)
**Rosaceae**	*Crataegus monogyna* Jacq.	Alic Yemisen	E.1194/M<br>E.1450/A	Frs/M<br>Lvs/M<br>Frs/A	Crataegus : sclereids, calcium oxalate, aleurone grains, fixed oil	A,M: Hypertension, diuretic, antidiarrhoeal, cardiotonic, stomachic, headache, heart stimulant (**)<br>A: Arteriosclerosis, sedative (*)	Infusion<br>Decoction	HDD (7)<br>C1 (3)
**Rosaceae**	**Cydonia oblonga* Miller	Ayva	E.1197/M	Sds/M<br>Lvs/M	Mucilage, arabinose, xylose, uronic acid derivates fixed oil, arabinose, xylose, uronic acid	A,M: Tranquillizer, preventive, pharyngotonsillitis, hypertension, liver disease, antitussive, antiseptic in skin disorders (**)	Infusion	BC (5)
**Rosaceae**	**Cerasus avium* (L.) Moench.	Kiraz	E.1382/M	Stalk/M	Photasium salts, tannin	A,M: Heart stimulant, depurative, stomachic, antirheumatic, digestive, urinary system disorders, obesity (***)	Decoction	BC (7)
**Rosaceae**	*P. spinosa* L.	Cakal erigi	E.1381/M	Sds/M	Tannin, oils, capsicum, capsaicin, citric acid, resin, vitamin E	A,M: Insectisit, stomachic, antidiarrhoeal, lactogogue, hearth stimulant, sphagitis, urinary system disorders, hypertension, alzheimer (***)	Decoction	BC (5)

**Rosaceae**	*Rosa canina* L.	Kusburnu	E.1195/M	Sds/M<br><br><br>Frs/A	Geraniol, citronellol (70-75 % alcohols), esters	A,M: Antidiarrhoeal, cardiotonic, hypoglycemian, diuretic, astringent, acidulous, obesity (***)<br>A: Grippe, stimulant, diabetes (***)	Decoction<br>Infusion	T (7)<br>KY (7)
**Rosaceae**	*Rubus canescens* D.C.	Tilkiuzumu<br>Bogurtlen	E.1378/M	Frs/M<br>Root/M<br>Lvs/M	Lectin, gums, glucoproteins, alkaloids, terpenoids, terpens	A,M: Stomatitis, diuretic, antilithic, depurative, hemagogue, hypertension, dysentery, antidiarrhoeal, hypoglycemian, antitussive, orexigenic, kidney stones (***)	Direct application<br>Decoction	T (7)<br>KY (5)
**Salicaceae**	**Salix babylonica* L.	Sogut	E.1397/M	Bark/M<br>Lvs/M<br>Root/M<br>Lvs/A	Salix spp. natural aspirin, glycosides, tannins	A,M: Stomachic, hepatoprotector, phymatosis, anodyne, antihemorrhagic (**)<br>A: Orexigenic, febrifuge (**)	Decoction	T (5)<br>C4 (5)<br>C2 (5)<br>KY (3)
**Solanaceae**	*Mandragora autumnalis* Bertol.	Adamotu	E.1398/M	Root/M<br>Stem/M<br>Flv/M<br><br>Root/A	Alkaloids, atropine, the fluorescent substance scopoletin	A,M: Aphrodisiac, analgesic, spazmolytic, narcotic, antidiarrhoeal, hypercholesterolemia (***)<br>A: Anodyne emetic, purgative, eczema (***)	Decoction	T (3)<br>NLH (7)<br>KY (7)<br>C1 (7)
**Solanaceae**	*Physalis alkekengi* L.	Guveyfeneri	E.1370/M	Sds/M	Chian turpentine	A,M: Diuretic, sudatorian, hypoglycemian (*).	Decoction	BC (3)

**Tiliaceae**	*Tilia argentea* Desf. ex. DC.	Ihlamur	E.1382/M	Flv/M<br>Lvs/M<br><br>Flv/A	Tilia spp. : Flavonoid, glycosides, mucilage, volatile oil, phenolic acids, proanthocyanidin	A,M: Respiratory system disorders, throat disorders, cardiotonic, cough (***)<br>A: Sedative, bronchial trouble, diuretic,<br>(***)	Decoction	C1 (9)<br>T (7)<br>KY (7)
**Urticaceae**	*Urtica urens* L.<br>*U. dioica* L.<br><br><br><br>*U. pilulifera* L.	Isirgan	E.1384/M<br><br><br><br><br>E.1438/A	Lvs/M<br>Stem/<br>M<br><br><br><br>Lvs/A<br>Sds/A<br>Root/<br>A	Photasium salts, vit. C, asetil colin	A,M: Antirrheumatic, cancer, asthma, tuberculosis, vulnerary, stomachic, liver disease, antihemorrhoidal, depurative, lactogogue, allergy, antiageing (***)<br>A: Diuretic,anticoagulant, antiseptic, goiter (***)	Decoction	C1 (7)<br>T (7)<br>NLH<br>(7)<br>KY (7)
**Valerianaceae**	*Valeriana officinalis* L.	Kediotu	E.1425/A	Root/<br>A	Volatile oil; esters, alcohols, ketone, alkaloids, valepotriates	A: Hypotension, sedative (*)	Decoction	C1 (3)
**Zygophyllaceae**	*Peganum harmala* L.	Uzerlik	E.1372/M	Sds/M	Indole alks	A,M: Emmenagogue, narcotic, sedative, diaphoretic, vermifuge, diuretic, nutritive, cephalalgia, antihysteria, notalgia, ophtalmalgia, omalgia, hand-tremble (**)	Vaporizatio<br>n	NLH<br>(5)<br>KY (5)
**Zygophyllaceae**	*Tribulus terrestris* L.	Deve cokerten<br>Demirdikeni<br><br>Coban cokerten<br>Yalanci akdiken	E.1380/M<br><br><br>E.1449/A<br>E.1460/A	Frs/M<br>Flv/M<br><br>Frs/A<br>Lvs/A	Saponin, diosgenin, amide, alkaloid, fixed oil, resin	A,M: Heart, blood stimulant, Arteriosclerosis (**)<br><br>A: Cardotonic, treat joandice (**)	Decoction	BC (7)

____

A: Adana, M: Mersin, C1-4: markets of Cerci Yusuf, KY: Kor Yusuf, NLH: Nursi Lokman Hekim, T: Tarsus, BC: Bilgehan Cetinkaya, HDD: Herderde deva
